# Terrestrial gastropods of Srebarna Nature Reserve, North-Eastern Bulgaria (Gastropoda)

**DOI:** 10.3897/BDJ.3.e4306

**Published:** 2015-01-20

**Authors:** Ivailo Dedov, Vera Antonova

**Affiliations:** † Institute of Biodiversity and Ecosystem Research at the Bulgarian Academy of Sciences, Sofia, Bulgaria

**Keywords:** Bulgaria, Srebarna Nature Reserve, terrestrial gastropods, fauna

## Abstract

We give the results from the first investigation focused on the land snail fauna in Srebarna Nature Reserve in Bulgaria. A total of 23 localities were studied and 27 species of terrestrial gastropods were found, 23 of which were new observations for the Reserve.

## Introduction

Srebarna Lake was declared as a protected site in 1942 and as a Nature Reserve in 1948. In 1998, according to the national nature protection legislation, it was classified as a “Managed Reserve”. In 1975, Srebarna Lake was also protected as a Wetland of International Importance under the Ramsar Convention and, in 1977, as a UNESCO Biosphere Reserve. In 1983, it was listed as World Natural and Cultural Heritage Site by UNESCO, and in 1989 the Lake was designated as European Important Bird Area (BG 033) by BirdLife International. Srebarna Lake was also included in Natura 2000 network in 2009 (http://www.ecolab.bas.bg, http://whc.unesco.org).

Srebarna is a hyper-euthrophic lake, located on the Bulgarian (right) bank of the Danube River between R.km 391 and R.km 393; UTM grid NJ 08. The Nature Reserve has a total surface area of 902.1 ha (Vasilev et al. 2012).

Until the recent study, no previous focused investigations had been carried out on terrestrial snails in the Reserve. Prokopich et al. (1970) reported the first land snails that functioned as intermediate hosts in their studies on cestodes: *Cepaea vindobonensis*, *Helix lucorum*, *Monacha cartusiana* and *Succinea putris*. Dedov (1998) found a total of seven species of land snails, adding three species to the previous observations: *Fruticicola fruticum*, *Chondrula tridens* and *Xerolenta obvia* (as *Helicella candicans* L. Pfeiffer, 1841).

## Materials and methods

In total, 23 localities were studied (Table 1). The terrestrial gastropods were collected by hand and soil samples were taken by I. Dedov (12 – 14.VI.2014) and V. Antonova (19 – 20.VII.2014). The material was stored in 70% ethanol or as dry shells. The species determination was done according to Damyanov and Likharev (1975) and Welter-Schultes (2012). The material is stored in the collection of I. Dedov in the Institute of Biodiversity and Ecosystem Research at the Bulgarian Academy of Sciences (IBER - BAS).

## Checklists

### Checklist of terrestrial gastropods

#### Acanthinula aculeata

(O.F. Muller 1774)

##### Materials

- **Type status:** Other material. **Occurrence:** sex: hermaphrodite; lifeStage: adult; **Taxon:** scientificName: *Acanthinula aculeata* (O.F. Muller 1774); kingdom: Animalia; phylum: Mollusca; class: Gastropoda; order: Pulmonata; family: Valloniidae; genus: Acanthinula; scientificNameAuthorship: (O.F. Muller 1774); **Location:** continent: Europe; country: Bulgaria; municipality: Silistra; locality: Srebarna Nature Reserve; locationRemarks: 11; decimalLatitude: 44.12875; decimalLongitude: 27.06027; georeferencedBy: Garmin 60 CS; **Identification:** identificationID: BG1620; **Event:** eventDate: 06-13-14; habitat: Deciduous forest

#### Aegopinella minor

(Stabile 1864)

##### Materials

- **Type status:** Other material. **Occurrence:** sex: hermaphrodite; lifeStage: adult; **Taxon:** scientificName: *Aegopinella minor* (Stabile 1864); kingdom: Animalia; phylum: Mollusca; class: Gastropoda; order: Pulmonata; family: Oxychilidae; genus: Aegopinella; scientificNameAuthorship: (Stabile 1864); **Location:** continent: Europe; country: Bulgaria; municipality: Silistra; locality: Srebarna Nature Reserve; locationRemarks: 19; decimalLatitude: 44.10249; decimalLongitude: 27.09153; georeferencedBy: Garmin 60 CS; **Identification:** identificationID: BG1628; **Event:** eventDate: 07-19-14; habitat: Deciduous forest
- **Type status:** Other material. **Occurrence:** sex: hermaphrodite; lifeStage: adult; **Taxon:** scientificName: *Aegopinella minor* (Stabile 1864); kingdom: Animalia; phylum: Mollusca; class: Gastropoda; order: Pulmonata; family: Oxychilidae; genus: Aegopinella; scientificNameAuthorship: (Stabile 1864); **Location:** continent: Europe; country: Bulgaria; municipality: Silistra; locality: Srebarna Nature Reserve; locationRemarks: 20; decimalLatitude: 44.10766; decimalLongitude: 27.08478; georeferencedBy: Garmin 60 CS; **Identification:** identificationID: BG1629; **Event:** eventDate: 07-19-14; habitat: Coniferous forest (Pinus nigra)

#### Cecilioides acicula

(O.F. Muller 1774)

##### Materials

- **Type status:** Other material. **Occurrence:** sex: hermaphrodite; lifeStage: adult; **Taxon:** scientificName: *Cecilioides acicula* (O.F. Muller 1774); kingdom: Animalia; phylum: Mollusca; class: Gastropoda; order: Pulmonata; family: Ferussaciidae; genus: Cecilioides; scientificNameAuthorship: (O.F. Muller 1774); **Location:** continent: Europe; country: Bulgaria; municipality: Silistra; locality: Srebarna Nature Reserve; locationRemarks: 11; decimalLatitude: 44.12875; decimalLongitude: 27.06027; georeferencedBy: Garmin 60 CS; **Identification:** identificationID: BG1620; **Event:** eventDate: 06-13-14; habitat: Deciduous forest

#### Cepaea vindobonensis

(C. Pfeiffer 1828)

##### Materials

- **Type status:** Other material. **Occurrence:** sex: hermaphrodite; lifeStage: adult; **Taxon:** scientificName: *Cepaea vindobonensis* (C. Pfeiffer 1828); kingdom: Animalia; phylum: Mollusca; class: Gastropoda; order: Pulmonata; family: Helicidae; genus: Cepaea; scientificNameAuthorship: (C. Pfeiffer 1828); **Location:** continent: Europe; country: Bulgaria; municipality: Silistra; locality: Srebarna Nature Reserve; locationRemarks: 2; decimalLatitude: 44.0992; decimalLongitude: 27.0648; georeferencedBy: Garmin 60 CS; **Identification:** identificationID: BG1611; **Event:** eventDate: 06-13-14; habitat: Yards and hedges
- **Type status:** Other material. **Occurrence:** sex: hermaphrodite; lifeStage: adult; **Taxon:** scientificName: *Cepaea vindobonensis* (C. Pfeiffer 1828); kingdom: Animalia; phylum: Mollusca; class: Gastropoda; order: Pulmonata; family: Helicidae; genus: Cepaea; scientificNameAuthorship: (C. Pfeiffer 1828); **Location:** continent: Europe; country: Bulgaria; municipality: Silistra; locality: Srebarna Nature Reserve; locationRemarks: 3; decimalLatitude: 44.09339; decimalLongitude: 27.06393; georeferencedBy: Garmin 60 CS; **Identification:** identificationID: BG1612; **Event:** eventDate: 06-15-14; habitat: meadows near roads in the village
- **Type status:** Other material. **Occurrence:** sex: hermaphrodite; lifeStage: adult; **Taxon:** scientificName: *Cepaea vindobonensis* (C. Pfeiffer 1828); kingdom: Animalia; phylum: Mollusca; class: Gastropoda; order: Pulmonata; family: Helicidae; genus: Cepaea; scientificNameAuthorship: (C. Pfeiffer 1828); **Location:** continent: Europe; country: Bulgaria; municipality: Silistra; locality: Srebarna Nature Reserve; locationRemarks: 5; decimalLatitude: 44.10302; decimalLongitude: 27.06354; georeferencedBy: Garmin 60 CS; **Identification:** identificationID: BG1614; **Event:** eventDate: 06-13-14; habitat: meadow near Srebarna lake
- **Type status:** Other material. **Occurrence:** sex: hermaphrodite; lifeStage: adult; **Taxon:** scientificName: *Cepaea vindobonensis* (C. Pfeiffer 1828); kingdom: Animalia; phylum: Mollusca; class: Gastropoda; order: Pulmonata; family: Helicidae; genus: Cepaea; scientificNameAuthorship: (C. Pfeiffer 1828); **Location:** continent: Europe; country: Bulgaria; municipality: Silistra; locality: Srebarna Nature Reserve; locationRemarks: 6; decimalLatitude: 44.10738; decimalLongitude: 27.05965; georeferencedBy: Garmin 60 CS; **Identification:** identificationID: BG1615; **Event:** eventDate: 06-13-14; habitat: meadows and bushes
- **Type status:** Other material. **Occurrence:** sex: hermaphrodite; lifeStage: adult; **Taxon:** scientificName: *Cepaea vindobonensis* (C. Pfeiffer 1828); kingdom: Animalia; phylum: Mollusca; class: Gastropoda; order: Pulmonata; family: Helicidae; genus: Cepaea; scientificNameAuthorship: (C. Pfeiffer 1828); **Location:** continent: Europe; country: Bulgaria; municipality: Silistra; locality: Srebarna Nature Reserve; locationRemarks: 7; decimalLatitude: 44.10758; decimalLongitude: 27.05853; georeferencedBy: Garmin 60 CS; **Identification:** identificationID: BG1616; **Event:** eventDate: 06-13-14; habitat: meadows and bushes
- **Type status:** Other material. **Occurrence:** sex: hermaphrodite; lifeStage: adult; **Taxon:** scientificName: *Cepaea vindobonensis* (C. Pfeiffer 1828); kingdom: Animalia; phylum: Mollusca; class: Gastropoda; order: Pulmonata; family: Helicidae; genus: Cepaea; scientificNameAuthorship: (C. Pfeiffer 1828); **Location:** continent: Europe; country: Bulgaria; municipality: Silistra; locality: Srebarna Nature Reserve; locationRemarks: 10; decimalLatitude: 44.13043; decimalLongitude: 27.06131; georeferencedBy: Garmin 60 CS; **Identification:** identificationID: BG1619; **Event:** eventDate: 06-13-14; habitat: open areas near Danube revier
- **Type status:** Other material. **Occurrence:** sex: hermaphrodite; lifeStage: adult; **Taxon:** scientificName: *Cepaea vindobonensis* (C. Pfeiffer 1828); kingdom: Animalia; phylum: Mollusca; class: Gastropoda; order: Pulmonata; family: Helicidae; genus: Cepaea; scientificNameAuthorship: (C. Pfeiffer 1828); **Location:** continent: Europe; country: Bulgaria; municipality: Silistra; locality: Srebarna Nature Reserve; locationRemarks: 11; decimalLatitude: 44.12875; decimalLongitude: 27.06027; georeferencedBy: Garmin 60 CS; **Identification:** identificationID: BG1620; **Event:** eventDate: 06-13-14; habitat: (Deciduous forest)
- **Type status:** Other material. **Occurrence:** sex: hermaphrodite; lifeStage: adult; **Taxon:** scientificName: *Cepaea vindobonensis* (C. Pfeiffer 1828); kingdom: Animalia; phylum: Mollusca; class: Gastropoda; order: Pulmonata; family: Helicidae; genus: Cepaea; scientificNameAuthorship: (C. Pfeiffer 1828); **Location:** continent: Europe; country: Bulgaria; municipality: Silistra; locality: Srebarna Nature Reserve; locationRemarks: 12; decimalLatitude: 44.08833; decimalLongitude: 27.06701; georeferencedBy: Garmin 60 CS; **Identification:** identificationID: BG1621; **Event:** eventDate: 06-14-14; habitat: meadows and bushes
- **Type status:** Other material. **Occurrence:** sex: hermaphrodite; lifeStage: adult; **Taxon:** scientificName: *Cepaea vindobonensis* (C. Pfeiffer 1828); kingdom: Animalia; phylum: Mollusca; class: Gastropoda; order: Pulmonata; family: Helicidae; genus: Cepaea; scientificNameAuthorship: (C. Pfeiffer 1828); **Location:** continent: Europe; country: Bulgaria; municipality: Silistra; locality: Srebarna Nature Reserve; locationRemarks: 13; decimalLatitude: 44.08865; decimalLongitude: 27.06411; georeferencedBy: Garmin 60 CS; **Identification:** identificationID: BG1622; **Event:** eventDate: 06-14-14; habitat: meadows and bushes
- **Type status:** Other material. **Occurrence:** sex: hermaphrodite; lifeStage: adult; **Taxon:** scientificName: *Cepaea vindobonensis* (C. Pfeiffer 1828); kingdom: Animalia; phylum: Mollusca; class: Gastropoda; order: Pulmonata; family: Helicidae; genus: Cepaea; scientificNameAuthorship: (C. Pfeiffer 1828); **Location:** continent: Europe; country: Bulgaria; municipality: Silistra; locality: Srebarna Nature Reserve; locationRemarks: 14; decimalLatitude: 44.08939; decimalLongitude: 27.07399; georeferencedBy: Garmin 60 CS; **Identification:** identificationID: BG1623; **Event:** eventDate: 06-14-14; habitat: meadows and bushes

##### Ecological interactions

###### Conservation status

IUCN: LC

#### Chondrula microtragus

(Rossmassler 1839)

##### Materials

- **Type status:** Other material. **Occurrence:** sex: hermaphrodite; lifeStage: adult; **Taxon:** scientificName: *Chondrula microtragus* (Rossmassler 1839); kingdom: Animalia; phylum: Mollusca; class: Gastropoda; order: Pulmonata; family: Enidae; genus: Chondrula; scientificNameAuthorship: (Rossmassler 1839); **Location:** continent: Europe; country: Bulgaria; municipality: Silistra; locality: Srebarna Nature Reserve; locationRemarks: 11; decimalLatitude: 44.12875; decimalLongitude: 27.06027; georeferencedBy: Garmin 60 CS; **Identification:** identificationID: BG1620; **Event:** eventDate: 06-13-14; habitat: Meadows and open areas
- **Type status:** Other material. **Occurrence:** sex: hermaphrodite; lifeStage: adult; **Taxon:** scientificName: *Chondrula microtragus* (Rossmassler 1839); kingdom: Animalia; phylum: Mollusca; class: Gastropoda; order: Pulmonata; family: Enidae; genus: Chondrula; scientificNameAuthorship: (Rossmassler 1839); **Location:** continent: Europe; country: Bulgaria; municipality: Silistra; locality: Srebarna Nature Reserve; locationRemarks: 18; decimalLatitude: 44.09999; decimalLongitude: 27.09350; georeferencedBy: Garmin 60 CS; **Identification:** identificationID: BG1627; **Event:** eventDate: 07-19-14; habitat: Meadows and open areas

##### Ecological interactions

###### Conservation status

IUCN: LC

#### Chondrula tridens

(O.F. Muller 1774)

##### Materials

- **Type status:** Other material. **Occurrence:** sex: hermaphrodite; lifeStage: adult; **Taxon:** scientificName: *Chondrula tridens* (O.F. Muller 1774); kingdom: Animalia; phylum: Mollusca; class: Gastropoda; order: Pulmonata; family: Enidae; genus: Chondrula; scientificNameAuthorship: (O.F. Muller 1774); **Location:** continent: Europe; country: Bulgaria; municipality: Silistra; locality: Srebarna Nature Reserve; locationRemarks: 1; decimalLatitude: 44.0949; decimalLongitude: 27.0639; georeferencedBy: Garmin 60 CS; **Identification:** identificationID: BG1610; **Event:** eventDate: 06-12-14; habitat: Yards and hedges, stone fence
- **Type status:** Other material. **Occurrence:** sex: hermaphrodite; lifeStage: adult; **Taxon:** scientificName: *Chondrula tridens* (O.F. Muller 1774); kingdom: Animalia; phylum: Mollusca; class: Gastropoda; order: Pulmonata; family: Enidae; genus: Chondrula; scientificNameAuthorship: (O.F. Muller 1774); **Location:** continent: Europe; country: Bulgaria; municipality: Silistra; locality: Srebarna Nature Reserve; locationRemarks: 15; decimalLatitude: 44.08937; decimalLongitude: 27.06819; georeferencedBy: Garmin 60 CS; **Identification:** identificationID: BG1624; **Event:** eventDate: 06-14-14; habitat: (Deciduous forest)
- **Type status:** Other material. **Occurrence:** sex: hermaphrodite; lifeStage: adult; **Taxon:** scientificName: *Chondrula tridens* (O.F. Muller 1774); kingdom: Animalia; phylum: Mollusca; class: Gastropoda; order: Pulmonata; family: Enidae; genus: Chondrula; scientificNameAuthorship: (O.F. Muller 1774); **Location:** continent: Europe; country: Bulgaria; municipality: Silistra; locality: Srebarna Nature Reserve; locationRemarks: 23; decimalLatitude: 44.09623; decimalLongitude: 27.07008; georeferencedBy: Garmin 60 CS; **Identification:** identificationID: BG1632; **Event:** eventDate: 07-20-14; habitat: meadows and open areas

#### Deroceras reticulatum

(O.F. Muller 1774)

##### Materials

- **Type status:** Other material. **Occurrence:** sex: hermaphrodite; lifeStage: adult; **Taxon:** scientificName: *Deroceras* af. *reticulatum* (O.F. Muller 1774); kingdom: Animalia; phylum: Mollusca; class: Gastropoda; order: Pulmonata; family: Agriolimacidae; genus: Deroceras; scientificNameAuthorship: (O.F. Muller 1774); **Location:** continent: Europe; country: Bulgaria; municipality: Silistra; locality: Srebarna Nature Reserve; locationRemarks: 2; decimalLatitude: 44.0992; decimalLongitude: 27.0648; georeferencedBy: Garmin 60 CS; **Identification:** identificationID: BG1611; **Event:** eventDate: 06-12-14; habitat: Yards and hedges

#### Helix pomatia

Linnaeus 1758

##### Materials

- **Type status:** Other material. **Occurrence:** sex: hermaphrodite; lifeStage: adult; **Taxon:** scientificName: *Helix pomatia* Linnaeus 1748; kingdom: Animalia; phylum: Mollusca; class: Gastropoda; order: Pulmonata; family: Helicidae; genus: Helix; scientificNameAuthorship: Linnaeus 1758; **Location:** continent: Europe; country: Bulgaria; municipality: Silistra; locality: Srebarna Nature Reserve; locationRemarks: 2; decimalLatitude: 44.0992; decimalLongitude: 27.0648; georeferencedBy: Garmin 60 CS; **Identification:** identificationID: BG1611; **Event:** eventDate: 06-12-14; habitat: Meadows and open areas,
- **Type status:** Other material. **Occurrence:** sex: hermaphrodite; lifeStage: adult; **Taxon:** scientificName: *Helix pomatia* Linnaeus 1748; kingdom: Animalia; phylum: Mollusca; class: Gastropoda; order: Pulmonata; family: Helicidae; genus: Helix; scientificNameAuthorship: Linnaeus 1758; **Location:** continent: Europe; country: Bulgaria; municipality: Silistra; locality: Srebarna Nature Reserve; locationRemarks: 3; decimalLatitude: 44.09339; decimalLongitude: 27.06393; georeferencedBy: Garmin 60 CS; **Identification:** identificationID: BG1612; **Event:** eventDate: 06-12-14; habitat: meadows near roads in the village
- **Type status:** Other material. **Occurrence:** sex: hermaphrodite; lifeStage: adult; **Taxon:** scientificName: *Helix pomatia* Linnaeus 1748; kingdom: Animalia; phylum: Mollusca; class: Gastropoda; order: Pulmonata; family: Helicidae; genus: Helix; scientificNameAuthorship: Linnaeus 1758; **Location:** continent: Europe; country: Bulgaria; municipality: Silistra; locality: Srebarna Nature Reserve; locationRemarks: 4; decimalLatitude: 44.10314; decimalLongitude: 27.06285; georeferencedBy: Garmin 60 CS; **Identification:** identificationID: BG1613; **Event:** eventDate: 06-13-14; habitat: meadows and bushes
- **Type status:** Other material. **Occurrence:** sex: hermaphrodite; lifeStage: adult; **Taxon:** scientificName: *Helix pomatia* Linnaeus 1748; kingdom: Animalia; phylum: Mollusca; class: Gastropoda; order: Pulmonata; family: Helicidae; genus: Helix; scientificNameAuthorship: Linnaeus 1758; **Location:** continent: Europe; country: Bulgaria; municipality: Silistra; locality: Srebarna Nature Reserve; locationRemarks: 5; decimalLatitude: 44.10302; decimalLongitude: 27.06354; georeferencedBy: Garmin 60 CS; **Identification:** identificationID: BG1614; **Event:** eventDate: 06-13-14; habitat: meadow near lake
- **Type status:** Other material. **Occurrence:** sex: hermaphrodite; lifeStage: adult; **Taxon:** scientificName: *Helix pomatia* Linnaeus 1748; kingdom: Animalia; phylum: Mollusca; class: Gastropoda; order: Pulmonata; family: Helicidae; genus: Helix; scientificNameAuthorship: Linnaeus 1758; **Location:** continent: Europe; country: Bulgaria; municipality: Silistra; locality: Srebarna Nature Reserve; locationRemarks: 6; decimalLatitude: 44.10738; decimalLongitude: 27.05965; georeferencedBy: Garmin 60 CS; **Identification:** identificationID: BG1615; **Event:** eventDate: 06-13-14; habitat: meadows and bushes
- **Type status:** Other material. **Occurrence:** sex: hermaphrodite; lifeStage: adult; **Taxon:** scientificName: *Helix pomatia* Linnaeus 1748; kingdom: Animalia; phylum: Mollusca; class: Gastropoda; order: Pulmonata; family: Helicidae; genus: Helix; scientificNameAuthorship: Linnaeus 1758; **Location:** continent: Europe; country: Bulgaria; municipality: Silistra; locality: Srebarna Nature Reserve; locationRemarks: 8; decimalLatitude: 44.11252; decimalLongitude: 27.05658; georeferencedBy: Garmin 60 CS; **Identification:** identificationID: BG1617; **Event:** eventDate: 06-13-14; habitat: meadows and bushes
- **Type status:** Other material. **Occurrence:** sex: hermaphrodite; lifeStage: adult; **Taxon:** scientificName: *Helix pomatia* Linnaeus 1748; kingdom: Animalia; phylum: Mollusca; class: Gastropoda; order: Pulmonata; family: Helicidae; genus: Helix; scientificNameAuthorship: Linnaeus 1758; **Location:** continent: Europe; country: Bulgaria; municipality: Silistra; locality: Srebarna Nature Reserve; locationRemarks: 10; decimalLatitude: 44.13043; decimalLongitude: 27.06131; georeferencedBy: Garmin 60 CS; **Identification:** identificationID: BG1619; **Event:** eventDate: 06-13-14; habitat: Meadows and open areas,
- **Type status:** Other material. **Occurrence:** sex: hermaphrodite; lifeStage: adult; **Taxon:** scientificName: *Helix pomatia* Linnaeus 1748; kingdom: Animalia; phylum: Mollusca; class: Gastropoda; order: Pulmonata; family: Helicidae; genus: Helix; scientificNameAuthorship: Linnaeus 1758; **Location:** continent: Europe; country: Bulgaria; municipality: Silistra; locality: Srebarna Nature Reserve; locationRemarks: 11; decimalLatitude: 44.12875; decimalLongitude: 27.06027; georeferencedBy: Garmin 60 CS; **Identification:** identificationID: BG1620; **Event:** eventDate: 06-13-14; habitat: Deciduous forest
- **Type status:** Other material. **Occurrence:** sex: hermaphrodite; lifeStage: adult; **Taxon:** scientificName: *Helix pomatia* Linnaeus 1748; kingdom: Animalia; phylum: Mollusca; class: Gastropoda; order: Pulmonata; family: Helicidae; genus: Helix; scientificNameAuthorship: Linnaeus 1758; **Location:** continent: Europe; country: Bulgaria; municipality: Silistra; locality: Srebarna Nature Reserve; locationRemarks: 12; decimalLatitude: 44.08833; decimalLongitude: 27.06701; georeferencedBy: Garmin 60 CS; **Identification:** identificationID: BG1621; **Event:** eventDate: 06-14-14; habitat: meadows and bushes
- **Type status:** Other material. **Occurrence:** sex: hermaphrodite; lifeStage: adult; **Taxon:** scientificName: *Helix pomatia* Linnaeus 1748; kingdom: Animalia; phylum: Mollusca; class: Gastropoda; order: Pulmonata; family: Helicidae; genus: Helix; scientificNameAuthorship: Linnaeus 1758; **Location:** continent: Europe; country: Bulgaria; municipality: Silistra; locality: Srebarna Nature Reserve; locationRemarks: 13; decimalLatitude: 44.08865; decimalLongitude: 27.06411; georeferencedBy: Garmin 60 CS; **Identification:** identificationID: BG1622; **Event:** eventDate: 06-14-14; habitat: meadows and bushes
- **Type status:** Other material. **Occurrence:** sex: hermaphrodite; lifeStage: adult; **Taxon:** scientificName: *Helix pomatia* Linnaeus 1748; kingdom: Animalia; phylum: Mollusca; class: Gastropoda; order: Pulmonata; family: Helicidae; genus: Helix; scientificNameAuthorship: Linnaeus 1758; **Location:** continent: Europe; country: Bulgaria; municipality: Silistra; locality: Srebarna Nature Reserve; locationRemarks: 14; decimalLatitude: 44.08939; decimalLongitude: 27.07399; georeferencedBy: Garmin 60 CS; **Identification:** identificationID: BG1623; **Event:** eventDate: 06-14-14; habitat: meadows and bushes

##### Ecological interactions

###### Conservation status

IUCN: LC, BSG, ZBR-41

#### Laciniaria plicata

(Draparnaud 1801)

##### Materials

- **Type status:** Other material. **Occurrence:** sex: hermaphrodite; lifeStage: adult; **Taxon:** scientificName: *Laciniaria plicata* (Draparnaud 1801); kingdom: Animalia; phylum: Mollusca; class: Gastropoda; order: Pulmonata; family: Clausiliidae; genus: Laciniaria; scientificNameAuthorship: (Draparnaud 1801); **Location:** continent: Europe; country: Bulgaria; municipality: Silistra; locality: Srebarna Nature Reserve; locationRemarks: 11; decimalLatitude: 44.12875; decimalLongitude: 27.06027; georeferencedBy: Garmin 60 CS; **Identification:** identificationID: BG1620; **Event:** eventDate: 06-13-14; habitat: Deciduous forest

#### Limacus flavus

(Linnaeus 1758)

##### Materials

- **Type status:** Other material. **Occurrence:** sex: hermaphrodite; lifeStage: adult; **Taxon:** scientificName: *Limacus flavus* (Linnaeus 1758); kingdom: Animalia; phylum: Mollusca; class: Gastropoda; order: Pulmonata; family: Limacidae; genus: Limacus; scientificNameAuthorship: (Linnaeus 1758); **Location:** continent: Europe; country: Bulgaria; municipality: Silistra; locality: Srebarna Nature Reserve; locationRemarks: 2; decimalLatitude: 44.0992; decimalLongitude: 27.0648; georeferencedBy: Garmin 60 CS; **Identification:** identificationID: BG1611; **Event:** eventDate: 06-12-14; habitat: Yards and hedges

#### Lindholmiola girva

(Frivaldsky 1835)

##### Materials

- **Type status:** Other material. **Occurrence:** sex: hermaphrodite; lifeStage: adult; **Taxon:** scientificName: *Lindholmiola girva* (Frivaldsky 1835); kingdom: Animalia; phylum: Mollusca; class: Gastropoda; order: Pulmonata; family: Helicodontidae; genus: Lindholmiola; scientificNameAuthorship: (Frivaldsky 1835); **Location:** continent: Europe; country: Bulgaria; municipality: Silistra; locality: Srebarna Nature Reserve; locationRemarks: 11; decimalLatitude: 44.12875; decimalLongitude: 27.06027; georeferencedBy: Garmin 60 CS; **Identification:** identificationID: BG1620; **Event:** eventDate: 06-13-14; habitat: Deciduous forest
- **Type status:** Other material. **Occurrence:** sex: hermaphrodite; lifeStage: adult; **Taxon:** scientificName: *Lindholmiola girva* (Frivaldsky 1835); kingdom: Animalia; phylum: Mollusca; class: Gastropoda; order: Pulmonata; family: Helicodontidae; genus: Lindholmiola; scientificNameAuthorship: (Frivaldsky 1835); **Location:** continent: Europe; country: Bulgaria; municipality: Silistra; locality: Srebarna Nature Reserve; locationRemarks: 19; decimalLatitude: 44.10249; decimalLongitude: 27.09153; georeferencedBy: Garmin 60 CS; **Identification:** identificationID: BG1628; **Event:** eventDate: 07-19-14; habitat: Deciduous forest

##### Ecological interactions

###### Conservation status

IUCN: LC

#### Monacha carascaloides

(Bourguignat 1855)

##### Materials

- **Type status:** Other material. **Occurrence:** sex: hermaphrodite; lifeStage: adult; **Taxon:** scientificName: *Monacha carascaloides* (Bourguignat 1855); kingdom: Animalia; phylum: Mollusca; class: Gastropoda; order: Pulmonata; family: Hygromiidae; genus: Monacha; scientificNameAuthorship: (Bourguignat 1855); **Location:** continent: Europe; country: Bulgaria; municipality: Silistra; locality: Srebarna Nature Reserve; locationRemarks: 2; decimalLatitude: 44.0992; decimalLongitude: 27.0648; georeferencedBy: Garmin 60 CS; **Identification:** identificationID: BG1611; **Event:** eventDate: 06-12-14; habitat: Meadows and open areas,
- **Type status:** Other material. **Occurrence:** sex: hermaphrodite; lifeStage: adult; **Taxon:** scientificName: *Monacha carascaloides* (Bourguignat 1855); kingdom: Animalia; phylum: Mollusca; class: Gastropoda; order: Pulmonata; family: Hygromiidae; genus: Monacha; scientificNameAuthorship: (Bourguignat 1855); **Location:** continent: Europe; country: Bulgaria; municipality: Silistra; locality: Srebarna Nature Reserve; locationRemarks: 3; decimalLatitude: 44.09339; decimalLongitude: 27.06393; georeferencedBy: Garmin 60 CS; **Identification:** identificationID: BG1612; **Event:** eventDate: 06-12-14; habitat: meadows near roads in the village
- **Type status:** Other material. **Occurrence:** sex: hermaphrodite; lifeStage: adult; **Taxon:** scientificName: *Monacha carascaloides* (Bourguignat 1855); kingdom: Animalia; phylum: Mollusca; class: Gastropoda; order: Pulmonata; family: Hygromiidae; genus: Monacha; scientificNameAuthorship: (Bourguignat 1855); **Location:** continent: Europe; country: Bulgaria; municipality: Silistra; locality: Srebarna Nature Reserve; locationRemarks: 4; decimalLatitude: 44.10314; decimalLongitude: 27.06285; georeferencedBy: Garmin 60 CS; **Identification:** identificationID: BG1613; **Event:** eventDate: 06-13-14; habitat: meadows and bushes
- **Type status:** Other material. **Occurrence:** sex: hermaphrodite; lifeStage: adult; **Taxon:** scientificName: *Monacha carascaloides* (Bourguignat 1855); kingdom: Animalia; phylum: Mollusca; class: Gastropoda; order: Pulmonata; family: Hygromiidae; genus: Monacha; scientificNameAuthorship: (Bourguignat 1855); **Location:** continent: Europe; country: Bulgaria; municipality: Silistra; locality: Srebarna Nature Reserve; locationRemarks: 5; decimalLatitude: 44.10302; decimalLongitude: 27.06354; georeferencedBy: Garmin 60 CS; **Identification:** identificationID: BG1614; **Event:** eventDate: 06-13-14; habitat: meadow near lake
- **Type status:** Other material. **Occurrence:** sex: hermaphrodite; lifeStage: adult; **Taxon:** scientificName: *Monacha carascaloides* (Bourguignat 1855); kingdom: Animalia; phylum: Mollusca; class: Gastropoda; order: Pulmonata; family: Hygromiidae; genus: Monacha; scientificNameAuthorship: (Bourguignat 1855); **Location:** continent: Europe; country: Bulgaria; municipality: Silistra; locality: Srebarna Nature Reserve; locationRemarks: 6; decimalLatitude: 44.10738; decimalLongitude: 27.05965; georeferencedBy: Garmin 60 CS; **Identification:** identificationID: BG1615; **Event:** eventDate: 06-13-14; habitat: meadows and bushes
- **Type status:** Other material. **Occurrence:** sex: hermaphrodite; lifeStage: adult; **Taxon:** scientificName: *Monacha carascaloides* (Bourguignat 1855); kingdom: Animalia; phylum: Mollusca; class: Gastropoda; order: Pulmonata; family: Hygromiidae; genus: Monacha; scientificNameAuthorship: (Bourguignat 1855); **Location:** continent: Europe; country: Bulgaria; municipality: Silistra; locality: Srebarna Nature Reserve; locationRemarks: 8; decimalLatitude: 44.11252; decimalLongitude: 27.05658; georeferencedBy: Garmin 60 CS; **Identification:** identificationID: BG1617; **Event:** eventDate: 06-13-14; habitat: meadows and bushes
- **Type status:** Other material. **Occurrence:** sex: hermaphrodite; lifeStage: adult; **Taxon:** scientificName: *Monacha carascaloides* (Bourguignat 1855); kingdom: Animalia; phylum: Mollusca; class: Gastropoda; order: Pulmonata; family: Hygromiidae; genus: Monacha; scientificNameAuthorship: (Bourguignat 1855); **Location:** continent: Europe; country: Bulgaria; municipality: Silistra; locality: Srebarna Nature Reserve; locationRemarks: 11; decimalLatitude: 44.12875; decimalLongitude: 27.06027; georeferencedBy: Garmin 60 CS; **Identification:** identificationID: BG1620; **Event:** eventDate: 06-13-14; habitat: Deciduous forest
- **Type status:** Other material. **Occurrence:** sex: hermaphrodite; lifeStage: adult; **Taxon:** scientificName: *Monacha carascaloides* (Bourguignat 1855); kingdom: Animalia; phylum: Mollusca; class: Gastropoda; order: Pulmonata; family: Hygromiidae; genus: Monacha; scientificNameAuthorship: (Bourguignat 1855); **Location:** continent: Europe; country: Bulgaria; municipality: Silistra; locality: Srebarna Nature Reserve; locationRemarks: 12; decimalLatitude: 44.08833; decimalLongitude: 27.06701; georeferencedBy: Garmin 60 CS; **Identification:** identificationID: BG1621; **Event:** eventDate: 06-14-14; habitat: meadows and bushes
- **Type status:** Other material. **Occurrence:** sex: hermaphrodite; lifeStage: adult; **Taxon:** scientificName: *Monacha carascaloides* (Bourguignat 1855); kingdom: Animalia; phylum: Mollusca; class: Gastropoda; order: Pulmonata; family: Hygromiidae; genus: Monacha; scientificNameAuthorship: (Bourguignat 1855); **Location:** continent: Europe; country: Bulgaria; municipality: Silistra; locality: Srebarna Nature Reserve; locationRemarks: 13; decimalLatitude: 44.08865; decimalLongitude: 27.06411; georeferencedBy: Garmin 60 CS; **Identification:** identificationID: BG1622; **Event:** eventDate: 06-14-14; habitat: meadows and bushes
- **Type status:** Other material. **Occurrence:** sex: hermaphrodite; lifeStage: adult; **Taxon:** scientificName: *Monacha carascaloides* (Bourguignat 1855); kingdom: Animalia; phylum: Mollusca; class: Gastropoda; order: Pulmonata; family: Hygromiidae; genus: Monacha; scientificNameAuthorship: (Bourguignat 1855); **Location:** continent: Europe; country: Bulgaria; municipality: Silistra; locality: Srebarna Nature Reserve; locationRemarks: 14; decimalLatitude: 44.08939; decimalLongitude: 27.07399; georeferencedBy: Garmin 60 CS; **Identification:** identificationID: BG1623; **Event:** eventDate: 06-14-14; habitat: meadows and bushes

##### Ecological interactions

###### Conservation status

IUCN: LC

#### Monachoides incarnates

(O.F. Muller 1774)

##### Materials

- **Type status:** Other material. **Occurrence:** sex: hermaphrodite; lifeStage: adult; **Taxon:** scientificName: *Monachoides incarnates* (O.F. Muller 1774); kingdom: Animalia; phylum: Mollusca; class: Gastropoda; order: Pulmonata; family: Hygromiidae; genus: Monachoides; scientificNameAuthorship: (O.F. Muller 1774); **Location:** continent: Europe; country: Bulgaria; municipality: Silistra; locality: Srebarna Nature Reserve; locationRemarks: 16; decimalLatitude: 44.08802; decimalLongitude: 27.06490; georeferencedBy: Garmin 60 CS; **Identification:** identificationID: BG1625; **Event:** eventDate: 07-19-14; habitat: (Reed with single Acacia sp. trees)

##### Ecological interactions

###### Conservation status

IUCN: LC

#### Oxychilus sp.

##### Materials

- **Type status:** Other material. **Occurrence:** sex: hermaphrodite; lifeStage: juvenile; **Taxon:** scientificName: *Oxychilus* sp.; kingdom: Animalia; phylum: Mollusca; class: Gastropoda; order: Pulmonata; family: Oxychilidae; genus: Oxychilus; **Location:** continent: Europe; country: Bulgaria; municipality: Silistra; locality: Srebarna Nature Reserve; locationRemarks: 19; decimalLatitude: 44.10249; decimalLongitude: 27.09153; georeferencedBy: Garmin 60 CS; **Identification:** identificationID: BG1628; **Event:** eventDate: 07-19-14; habitat: Deciduous forest

#### Oxyloma elegans

(Risso 1826)

##### Materials

- **Type status:** Other material. **Occurrence:** sex: hermaphrodite; lifeStage: adult; **Taxon:** scientificName: Oxyloma cf. elegans (Risso 1826); kingdom: Animalia; phylum: Mollusca; class: Gastropoda; order: Pulmonata; family: Succineidae; genus: Oxyloma; scientificNameAuthorship: (Risso 1826); **Location:** continent: Europe; country: Bulgaria; municipality: Silistra; locality: Srebarna Nature Reserve; locationRemarks: 11; decimalLatitude: 44.12875; decimalLongitude: 27.06027; georeferencedBy: Garmin 60 CS; **Identification:** identificationID: BG1620; **Event:** eventDate: 06-13-14; habitat: (Deciduous forest)
- **Type status:** Other material. **Occurrence:** sex: hermaphrodite; lifeStage: adult; **Taxon:** scientificName: Oxyloma cf. elegans (Risso 1826); kingdom: Animalia; phylum: Mollusca; class: Gastropoda; order: Pulmonata; family: Succineidae; genus: Oxyloma; scientificNameAuthorship: (Risso 1826); **Location:** continent: Europe; country: Bulgaria; municipality: Silistra; locality: Srebarna Nature Reserve; locationRemarks: 16; decimalLatitude: 44.08802; decimalLongitude: 27.06490; georeferencedBy: Garmin 60 CS; **Identification:** identificationID: BG1625; **Event:** eventDate: 07-19-14; habitat: Reed with single Acacia sp. trees
- **Type status:** Other material. **Occurrence:** sex: hermaphrodite; lifeStage: adult; **Taxon:** scientificName: Oxyloma cf. elegans (Risso 1826); kingdom: Animalia; phylum: Mollusca; class: Gastropoda; order: Pulmonata; family: Succineidae; genus: Oxyloma; scientificNameAuthorship: (Risso 1826); **Location:** continent: Europe; country: Bulgaria; municipality: Silistra; locality: Srebarna Nature Reserve; locationRemarks: 22; decimalLatitude: 44.13167; decimalLongitude: 27.08780; georeferencedBy: Garmin 60 CS; **Identification:** identificationID: BG1631; **Event:** eventDate: 07-19-14; habitat: Danube river’s bank

#### Pomatias elegans

(O.F. Muller 1774)

##### Materials

- **Type status:** Other material. **Occurrence:** lifeStage: adult; **Taxon:** scientificName: *Pomatias elegans* (O.F. Muller 1774); kingdom: Animalia; phylum: Mollusca; class: Gastropoda; order: Neotaenioglossa; family: Pomatiidae; genus: Pomatias; scientificNameAuthorship: (O.F. Muller 1774); **Location:** continent: Europe; country: Bulgaria; municipality: Silistra; locality: Srebarna Nature Reserve; locationRemarks: 11; decimalLatitude: 44.12875; decimalLongitude: 27.06027; georeferencedBy: Garmin 60 CS; **Identification:** identificationID: BG1620; **Event:** eventDate: 06-13-14; habitat: Deciduous forest
- **Type status:** Other material. **Occurrence:** lifeStage: adult; **Taxon:** scientificName: *Pomatias elegans* (O.F. Muller 1774); kingdom: Animalia; phylum: Mollusca; class: Gastropoda; order: Neotaenioglossa; family: Pomatiidae; genus: Pomatias; scientificNameAuthorship: (O.F. Muller 1774); **Location:** continent: Europe; country: Bulgaria; municipality: Silistra; locality: Srebarna Nature Reserve; locationRemarks: 17; decimalLatitude: 44.09689; decimalLongitude: 27.09480; georeferencedBy: Garmin 60 CS; **Identification:** identificationID: BG1626; **Event:** eventDate: 07-19-14; habitat: Bushes in the ecotone of deciduous forest

#### Punctum pygmaeum

(Draparnaud 1801)

##### Materials

- **Type status:** Other material. **Occurrence:** sex: hermaphrodite; lifeStage: adult; **Taxon:** scientificName: *Punctum pygmaeum* (Draparnaud 1801); kingdom: Animalia; phylum: Mollusca; class: Gastropoda; order: Pulmonata; family: Punctidae; genus: Punctum; scientificNameAuthorship: (Draparnaud 1801); **Location:** continent: Europe; country: Bulgaria; municipality: Silistra; locality: Srebarna Nature Reserve; locationRemarks: 11; decimalLatitude: 44.12875; decimalLongitude: 27.06027; georeferencedBy: Garmin 60 CS; **Identification:** identificationID: BG1620; **Event:** eventDate: 06-13-14; habitat: Deciduous forest

#### Tandonia kusceri

(H. Wagner 1931)

##### Materials

- **Type status:** Other material. **Occurrence:** sex: hermaphrodite; lifeStage: adult; **Taxon:** scientificName: *Tandonia* af. *kusceri* (H. Wagner 1931); kingdom: Animalia; phylum: Mollusca; class: Gastropoda; order: Pulmonata; family: Milacidae; genus: Tandonia; scientificNameAuthorship: (H. Wagner 1931); **Location:** continent: Europe; country: Bulgaria; municipality: Silistra; locality: Srebarna Nature Reserve; locationRemarks: 2; decimalLatitude: 44.0992; decimalLongitude: 27.0648; georeferencedBy: Garmin 60 CS; **Identification:** identificationID: BG1611; **Event:** eventDate: 06-12-14; habitat: Yards and hedges
- **Type status:** Other material. **Occurrence:** sex: hermaphrodite; lifeStage: adult; **Taxon:** scientificName: *Tandonia* af. *kusceri* (H. Wagner 1931); kingdom: Animalia; phylum: Mollusca; class: Gastropoda; order: Pulmonata; family: Milacidae; genus: Tandonia; scientificNameAuthorship: (H. Wagner 1931); **Location:** continent: Europe; country: Bulgaria; municipality: Silistra; locality: Srebarna Nature Reserve; locationRemarks: 15; decimalLatitude: 44.08937; decimalLongitude: 27.06819; georeferencedBy: Garmin 60 CS; **Identification:** identificationID: BG1624; **Event:** eventDate: 06-14-14; habitat: (Deciduous forest)
- **Type status:** Other material. **Occurrence:** sex: hermaphrodite; lifeStage: adult; **Taxon:** scientificName: *Tandonia* af. *kusceri* (H. Wagner 1931); kingdom: Animalia; phylum: Mollusca; class: Gastropoda; order: Pulmonata; family: Milacidae; genus: Tandonia; scientificNameAuthorship: (H. Wagner 1931); **Location:** continent: Europe; country: Bulgaria; municipality: Silistra; locality: Srebarna Nature Reserve; locationRemarks: 16; decimalLatitude: 44.08802; decimalLongitude: 27.06490; georeferencedBy: Garmin 60 CS; **Identification:** identificationID: BG1625; **Event:** eventDate: 07-19-14; habitat: Reed with single Acacia sp. trees

#### Trochulus hispidus

(Linnaeus 1758)

##### Materials

- **Type status:** Other material. **Occurrence:** sex: hermaphrodite; lifeStage: adult; **Taxon:** scientificName: *Trochulus hispidus* (Linnaeus 1758); kingdom: Animalia; phylum: Mollusca; class: Gastropoda; order: Pulmonata; family: Hygromiidae; genus: Trochulus; scientificNameAuthorship: (Linnaeus 1758); **Location:** continent: Europe; country: Bulgaria; municipality: Silistra; locality: Srebarna Nature Reserve; locationRemarks: 11; decimalLatitude: 44.12875; decimalLongitude: 27.06027; georeferencedBy: Garmin 60 CS; **Identification:** identificationID: BG1620; **Event:** eventDate: 06-13-14; habitat: Deciduous forest

##### Ecological interactions

###### Conservation status

IUCN: LC

#### Truncatellina cylindrica

(A. Ferussac 1807)

##### Materials

- **Type status:** Other material. **Occurrence:** sex: hermaphrodite; lifeStage: adult; **Taxon:** scientificName: *Truncatellina cylindrica* (A. Ferussac 1807); kingdom: Animalia; phylum: Mollusca; class: Gastropoda; order: Pulmonata; family: Vertiginidae; genus: Truncatellina; scientificNameAuthorship: (A. Ferussac 1807); **Location:** continent: Europe; country: Bulgaria; municipality: Silistra; locality: Srebarna Nature Reserve; locationRemarks: 11; decimalLatitude: 44.12875; decimalLongitude: 27.06027; georeferencedBy: Garmin 60 CS; **Identification:** identificationID: BG1620; **Event:** eventDate: 06-13-14; habitat: Deciduous forest
- **Type status:** Other material. **Occurrence:** sex: hermaphrodite; lifeStage: adult; **Taxon:** scientificName: *Truncatellina cylindrica* (A. Ferussac 1807); kingdom: Animalia; phylum: Mollusca; class: Gastropoda; order: Pulmonata; family: Vertiginidae; genus: Truncatellina; scientificNameAuthorship: (A. Ferussac 1807); **Location:** continent: Europe; country: Bulgaria; municipality: Silistra; locality: Srebarna Nature Reserve; locationRemarks: 15; decimalLatitude: 44.08937; decimalLongitude: 27.06819; georeferencedBy: Garmin 60 CS; **Identification:** identificationID: BG1624; **Event:** eventDate: 06-14-14; habitat: Deciduous forest

#### Truncatellina rothi

(Reinhardt 1916)

##### Materials

- **Type status:** Other material. **Occurrence:** sex: hermaphrodite; lifeStage: adult; **Taxon:** scientificName: *Truncatellina rothi* (Reinhardt 1916); kingdom: Animalia; phylum: Mollusca; class: Gastropoda; order: Pulmonata; family: Vertiginidae; genus: Truncatellina; scientificNameAuthorship: (Reinhardt 1916); **Location:** continent: Europe; country: Bulgaria; municipality: Silistra; locality: Srebarna Nature Reserve; locationRemarks: 11; decimalLatitude: 44.12875; decimalLongitude: 27.06027; georeferencedBy: Garmin 60 CS; **Identification:** identificationID: BG1620; **Event:** eventDate: 06-13-14; habitat: Deciduous forest

##### Ecological interactions

###### Conservation status

IUCN: LC

#### Vallonia costata

(O.F. Muller 1774)

##### Materials

- **Type status:** Other material. **Occurrence:** sex: hermaphrodite; lifeStage: adult; **Taxon:** scientificName: *Vallonia costata* (O.F. Muller 1774); kingdom: Animalia; phylum: Mollusca; class: Gastropoda; order: Pulmonata; family: Valloniidae; genus: Vallonia; scientificNameAuthorship: (O.F. Muller 1774); **Location:** continent: Europe; country: Bulgaria; municipality: Silistra; locality: Srebarna Nature Reserve; locationRemarks: 11; decimalLatitude: 44.12875; decimalLongitude: 27.06027; georeferencedBy: Garmin 60 CS; **Identification:** identificationID: BG1620; **Event:** eventDate: 06-13-14; habitat: (Deciduous forest)
- **Type status:** Other material. **Occurrence:** sex: hermaphrodite; lifeStage: adult; **Taxon:** scientificName: *Vallonia costata* (O.F. Muller 1774); kingdom: Animalia; phylum: Mollusca; class: Gastropoda; order: Pulmonata; family: Valloniidae; genus: Vallonia; scientificNameAuthorship: (O.F. Muller 1774); **Location:** continent: Europe; country: Bulgaria; municipality: Silistra; locality: Srebarna Nature Reserve; locationRemarks: 15; decimalLatitude: 44.08937; decimalLongitude: 27.06819; georeferencedBy: Garmin 60 CS; **Identification:** identificationID: BG1624; **Event:** eventDate: 06-14-14; habitat: (Deciduous forest)

#### Vallonia enniensis

(Gredler 1856)

##### Materials

- **Type status:** Other material. **Occurrence:** sex: hermaphrodite; lifeStage: adult; **Taxon:** scientificName: *Vallonia enniensis* (Gredler 1856); kingdom: Animalia; phylum: Mollusca; class: Gastropoda; order: Pulmonata; family: Valloniidae; genus: Vallonia; scientificNameAuthorship: (Gredler 1856); **Location:** continent: Europe; country: Bulgaria; municipality: Silistra; locality: Srebarna Nature Reserve; locationRemarks: 11; decimalLatitude: 44.12875; decimalLongitude: 27.06027; georeferencedBy: Garmin 60 CS; **Identification:** identificationID: BG1620; **Event:** eventDate: 06-13-14; habitat: (Deciduous forest)

##### Ecological interactions

###### Conservation status

IUCN: DD

#### Vallonia excentrica

Sterki 1893

##### Materials

- **Type status:** Other material. **Occurrence:** sex: hermaphrodite; lifeStage: adult; **Taxon:** scientificName: *Vallonia excentrica* Sterki 1893; kingdom: Animalia; phylum: Mollusca; class: Gastropoda; order: Pulmonata; family: Valloniidae; genus: Vallonia; scientificNameAuthorship: Sterki 1893; **Location:** continent: Europe; country: Bulgaria; municipality: Silistra; locality: Srebarna Nature Reserve; locationRemarks: 11; decimalLatitude: 44.12875; decimalLongitude: 27.06027; georeferencedBy: Garmin 60 CS; **Identification:** identificationID: BG1620; **Event:** eventDate: 06-13-14; habitat: (Deciduous forest)
- **Type status:** Other material. **Occurrence:** sex: hermaphrodite; lifeStage: adult; **Taxon:** scientificName: *Vallonia excentrica* Sterki 1893; kingdom: Animalia; phylum: Mollusca; class: Gastropoda; order: Pulmonata; family: Valloniidae; genus: Vallonia; scientificNameAuthorship: Sterki 1893; **Location:** continent: Europe; country: Bulgaria; municipality: Silistra; locality: Srebarna Nature Reserve; locationRemarks: 15; decimalLatitude: 44.08937; decimalLongitude: 27.06819; georeferencedBy: Garmin 60 CS; **Identification:** identificationID: BG1624; **Event:** eventDate: 06-14-14; habitat: (Deciduous forest)

#### Vallonia pulchella

(O.F. Muller 1774)

##### Materials

- **Type status:** Other material. **Occurrence:** sex: hermaphrodite; lifeStage: adult; **Taxon:** scientificName: *Vallonia pulchella* (O.F. Muller 1774); kingdom: Animalia; phylum: Mollusca; class: Gastropoda; order: Pulmonata; family: Valloniidae; genus: Vallonia; scientificNameAuthorship: (O.F. Muller 1774); **Location:** continent: Europe; country: Bulgaria; municipality: Silistra; locality: Srebarna Nature Reserve; locationRemarks: 11; decimalLatitude: 44.12875; decimalLongitude: 27.06027; georeferencedBy: Garmin 60 CS; **Identification:** identificationID: BG1620; **Event:** eventDate: 06-13-14; habitat: (Deciduous forest)
- **Type status:** Other material. **Occurrence:** sex: hermaphrodite; lifeStage: adult; **Taxon:** scientificName: *Vallonia pulchella* (O.F. Muller 1774); kingdom: Animalia; phylum: Mollusca; class: Gastropoda; order: Pulmonata; family: Valloniidae; genus: Vallonia; scientificNameAuthorship: (O.F. Muller 1774); **Location:** continent: Europe; country: Bulgaria; municipality: Silistra; locality: Srebarna Nature Reserve; locationRemarks: 15; decimalLatitude: 44.08937; decimalLongitude: 27.06819; georeferencedBy: Garmin 60 CS; **Identification:** identificationID: BG1624; **Event:** eventDate: 06-14-14; habitat: (Deciduous forest)

#### Xerolenta obvia

(Menke 1828)

##### Materials

- **Type status:** Other material. **Occurrence:** sex: hermaphrodite; lifeStage: adult; **Taxon:** scientificName: *Xerolenta obvia* (Menke 1828); kingdom: Animalia; phylum: Mollusca; class: Gastropoda; order: Pulmonata; family: Hygromiidae; genus: Xerolenta; scientificNameAuthorship: (Menke 1828); **Location:** continent: Europe; country: Bulgaria; municipality: Silistra; locality: Srebarna Nature Reserve; locationRemarks: 2; decimalLatitude: 44.0992; decimalLongitude: 27.0648; georeferencedBy: Garmin 60 CS; **Identification:** identificationID: BG1611; **Event:** eventDate: 06-12-14; habitat: Meadows and open areas,
- **Type status:** Other material. **Occurrence:** sex: hermaphrodite; lifeStage: adult; **Taxon:** scientificName: *Xerolenta obvia* (Menke 1828); kingdom: Animalia; phylum: Mollusca; class: Gastropoda; order: Pulmonata; family: Hygromiidae; genus: Xerolenta; scientificNameAuthorship: (Menke 1828); **Location:** continent: Europe; country: Bulgaria; municipality: Silistra; locality: Srebarna Nature Reserve; locationRemarks: 3; decimalLatitude: 44.09339; decimalLongitude: 27.06393; georeferencedBy: Garmin 60 CS; **Identification:** identificationID: BG1612; **Event:** eventDate: 06-12-14; habitat: meadows near roads in the village
- **Type status:** Other material. **Occurrence:** sex: hermaphrodite; lifeStage: adult; **Taxon:** scientificName: *Xerolenta obvia* (Menke 1828); kingdom: Animalia; phylum: Mollusca; class: Gastropoda; order: Pulmonata; family: Hygromiidae; genus: Xerolenta; scientificNameAuthorship: (Menke 1828); **Location:** continent: Europe; country: Bulgaria; municipality: Silistra; locality: Srebarna Nature Reserve; locationRemarks: 4; decimalLatitude: 44.10314; decimalLongitude: 27.06285; georeferencedBy: Garmin 60 CS; **Identification:** identificationID: BG1613; **Event:** eventDate: 06-13-14; habitat: meadows and bushes
- **Type status:** Other material. **Occurrence:** sex: hermaphrodite; lifeStage: adult; **Taxon:** scientificName: *Xerolenta obvia* (Menke 1828); kingdom: Animalia; phylum: Mollusca; class: Gastropoda; order: Pulmonata; family: Hygromiidae; genus: Xerolenta; scientificNameAuthorship: (Menke 1828); **Location:** continent: Europe; country: Bulgaria; municipality: Silistra; locality: Srebarna Nature Reserve; locationRemarks: 11; decimalLatitude: 44.12875; decimalLongitude: 27.06027; georeferencedBy: Garmin 60 CS; **Identification:** identificationID: BG1620; **Event:** eventDate: 06-13-14; habitat: (Deciduous forest)
- **Type status:** Other material. **Occurrence:** sex: hermaphrodite; lifeStage: adult; **Taxon:** scientificName: *Xerolenta obvia* (Menke 1828); kingdom: Animalia; phylum: Mollusca; class: Gastropoda; order: Pulmonata; family: Hygromiidae; genus: Xerolenta; scientificNameAuthorship: (Menke 1828); **Location:** continent: Europe; country: Bulgaria; municipality: Silistra; locality: Srebarna Nature Reserve; locationRemarks: 12; decimalLatitude: 44.08833; decimalLongitude: 27.06701; georeferencedBy: Garmin 60 CS; **Identification:** identificationID: BG1621; **Event:** eventDate: 06-14-14; habitat: meadows and bushes
- **Type status:** Other material. **Occurrence:** sex: hermaphrodite; lifeStage: adult; **Taxon:** scientificName: *Xerolenta obvia* (Menke 1828); kingdom: Animalia; phylum: Mollusca; class: Gastropoda; order: Pulmonata; family: Hygromiidae; genus: Xerolenta; scientificNameAuthorship: (Menke 1828); **Location:** continent: Europe; country: Bulgaria; municipality: Silistra; locality: Srebarna Nature Reserve; locationRemarks: 13; decimalLatitude: 44.08865; decimalLongitude: 27.06411; georeferencedBy: Garmin 60 CS; **Identification:** identificationID: BG1622; **Event:** eventDate: 06-14-14; habitat: meadows and bushes
- **Type status:** Other material. **Occurrence:** sex: hermaphrodite; lifeStage: adult; **Taxon:** scientificName: *Xerolenta obvia* (Menke 1828); kingdom: Animalia; phylum: Mollusca; class: Gastropoda; order: Pulmonata; family: Hygromiidae; genus: Xerolenta; scientificNameAuthorship: (Menke 1828); **Location:** continent: Europe; country: Bulgaria; municipality: Silistra; locality: Srebarna Nature Reserve; locationRemarks: 14; decimalLatitude: 44.08939; decimalLongitude: 27.07399; georeferencedBy: Garmin 60 CS; **Identification:** identificationID: BG1623; **Event:** eventDate: 06-14-14; habitat: meadows and bushes

#### Zebrina detrita

(O.F. Muller 1774)

##### Materials

- **Type status:** Other material. **Occurrence:** sex: hermaphrodite; lifeStage: adult; **Taxon:** scientificName: *Zebrina detrita* (O.F. Muller 1774); kingdom: Animalia; phylum: Mollusca; class: Gastropoda; order: Pulmonata; family: Enidae; genus: Zebrina; scientificNameAuthorship: (O.F. Muller 1774); **Location:** continent: Europe; country: Bulgaria; municipality: Silistra; locality: Srebarna Nature Reserve; locationRemarks: 9; decimalLatitude: 44.12067; decimalLongitude: 27.06315; georeferencedBy: Garmin 60 CS; **Identification:** identificationID: BG1618; **Event:** eventDate: 06-13-14; habitat: meadows and open areas
- **Type status:** Other material. **Occurrence:** sex: hermaphrodite; lifeStage: adult; **Taxon:** scientificName: *Zebrina detrita* (O.F. Muller 1774); kingdom: Animalia; phylum: Mollusca; class: Gastropoda; order: Pulmonata; family: Enidae; genus: Zebrina; scientificNameAuthorship: (O.F. Muller 1774); **Location:** continent: Europe; country: Bulgaria; municipality: Silistra; locality: Srebarna Nature Reserve; locationRemarks: 11; decimalLatitude: 44.12875; decimalLongitude: 27.06027; georeferencedBy: Garmin 60 CS; **Identification:** identificationID: BG1620; **Event:** eventDate: 06-13-14; habitat: (Deciduous forest)
- **Type status:** Other material. **Occurrence:** sex: hermaphrodite; lifeStage: adult; **Taxon:** scientificName: *Zebrina detrita* (O.F. Muller 1774); kingdom: Animalia; phylum: Mollusca; class: Gastropoda; order: Pulmonata; family: Enidae; genus: Zebrina; scientificNameAuthorship: (O.F. Muller 1774); **Location:** continent: Europe; country: Bulgaria; municipality: Silistra; locality: Srebarna Nature Reserve; locationRemarks: 21; decimalLatitude: 44.11780; decimalLongitude: 27.0797; georeferencedBy: Garmin 60 CS; **Identification:** identificationID: BG1630; **Event:** eventDate: 07-19-14; habitat: Bushes

## Discussion

**Figure 1.**
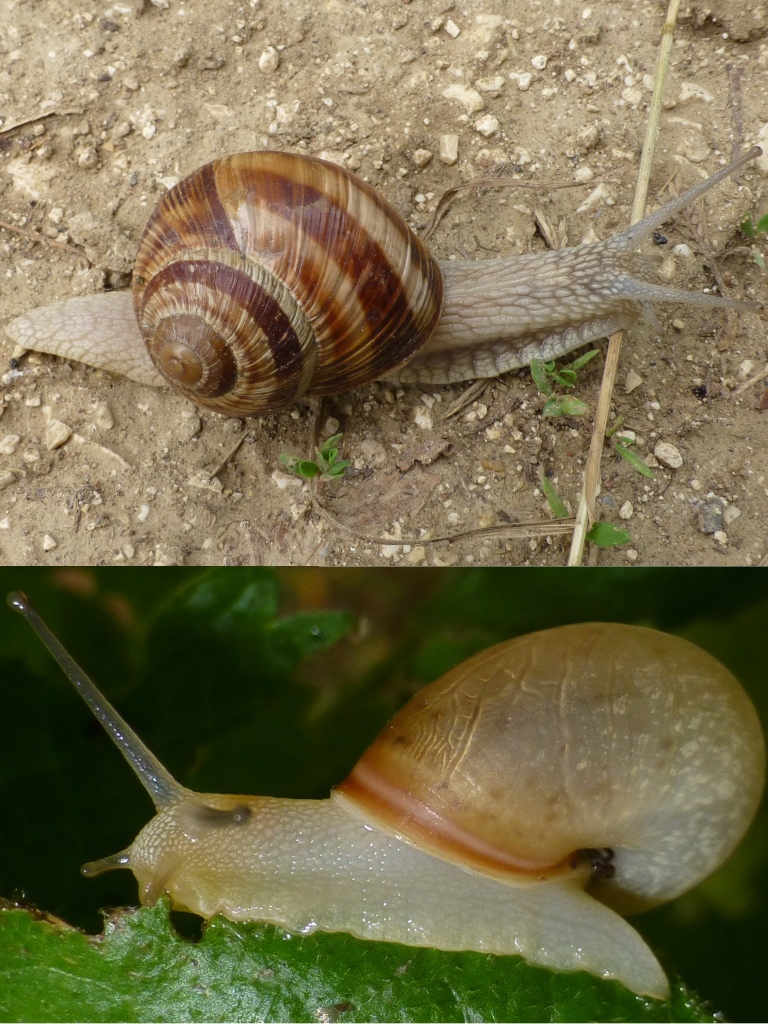
Above: *Helix pomatia* Linnaeus 1758 (diameter of shell 41 mm); below: *Monacha carascaloides* (Bourguignat 1855) (diameter of shell 16 mm), Sreburna reserve, buffer zone, meadow and bushes.

During the recent research in the Srebarna protected area, 27 land snails species were found: 23 of them were new species records for the reserve and four had been documented in the area previously. *F. fruticum* (reported by Dedov 1998) and *H. lucorum* (published by Prokopich et al. 1970) were not found. Anatomical and conchological investigation of specimens identified as *Monacha*, and which fit the description of *M. cartusiana* of Prokopich et al. (1970), showed the specimens to be *M. carascaloides* (Fig. 1).

The species Oxyloma cf. elegans needs further confirmation by anatomical characteristics. During the field work, adult specimens were found only as empty shells. According to the key of Damyanov and Likharev (1975), the height of the mature shells (about 22.5 мм) corresponds to *S. putris* (see Dedov 1998), but the body color of the single juvenile specimen found alive is dark-patched pigmented as in the genus *Oxyloma* (Welter-Schultes 2012). Most probably both Oxyloma cf. elegans and *S. putris* occur in the reserve.

The species found in most localities were *Helix pomatia* (11 sites, Fig. 1), *C. vindobonensis* (10 sites), *M. carascaloides* (10 sites) and *X. obvia* (7 sites). The recorded species belong to 15 families, the most species rich of which are Valloniidae (5 species), Hygromiidae (4) and Enidae (3).

Of all the species recorded, eight have been evaluated in the “IUCN red list of threatened species 2014” to belong to the category LC (Least Concern): *C. vindobonensis*, *Ch. microtragus*, *H. pomatia*, *L. girva*, *M. incarnatus*, *M. carascaloides*, *T. hispidus*, *T. rothi* and one - (*V. enniensis*) - in the category DD (Data Deficient). Another two species (*H. lucorum* and *H. pomatia*) are mentioned in the Bulgarian conservation legislation, as they are included in Section III (Regulated use of plant and animal species), Art. 41 (1), Annex 4 of “Biodiversity Act” (State Gazette 2002).

The deciduous forests in the buffer zone of Srebarna Reserve were found to be richer in gastropod species than the reserve itself (a total of 24 species, 13 of which were found only in this habitat). These were meso-xerophyle and xerophyle species which live in the forest ecotone (*C. vindobonensis*, *Ch. microtragus*, *Ch. tridens*, *M. carascaloides*, *V. costata*, *V. excentrica*, *V. pulchella*, *X. obvia*, *Z. detrita*) or meso-hygrophyle and hygrophyle species, occasionally found into the samples (*V. enniensis*, Succineidae). Eight species of conservation significance were found in the deciduous forests (*C. vindobonensis*, *Ch. microtragus*, *H. pomatia*, *L. girva*, *M. carascaloides*, *M. incarnatus*, *Tr. hispidus* and *Tr. rothi*).

In the open and urban areas, nine species were found and two of them were of conservation significance (*C. vindobonensis* and *H. pomatia*). The slug D. cf. reticulatum was found only in open habitats.

## Supplementary Material

XML Treatment for Acanthinula aculeata

XML Treatment for Aegopinella minor

XML Treatment for Cecilioides acicula

XML Treatment for Cepaea vindobonensis

XML Treatment for Chondrula microtragus

XML Treatment for Chondrula tridens

XML Treatment for Deroceras reticulatum

XML Treatment for Helix pomatia

XML Treatment for Laciniaria plicata

XML Treatment for Limacus flavus

XML Treatment for Lindholmiola girva

XML Treatment for Monacha carascaloides

XML Treatment for Monachoides incarnates

XML Treatment for Oxychilus sp.

XML Treatment for Oxyloma elegans

XML Treatment for Pomatias elegans

XML Treatment for Punctum pygmaeum

XML Treatment for Tandonia kusceri

XML Treatment for Trochulus hispidus

XML Treatment for Truncatellina cylindrica

XML Treatment for Truncatellina rothi

XML Treatment for Vallonia costata

XML Treatment for Vallonia enniensis

XML Treatment for Vallonia excentrica

XML Treatment for Vallonia pulchella

XML Treatment for Xerolenta obvia

XML Treatment for Zebrina detrita

## Figures and Tables

**Table 1.** Studied localities in Srebarna Nature Reserve.

**№**	**Coordinates**	**Locality**	**Date**	**Species**
1	44.0949 27.0639	village Srebarna, on stone fence	12-13.06.2014	*Chondrula tridens*
2	44.0992 27.0648	village Srebarna, yard of IBER house	12-15.06.2014	*Cepaea vindobonensis*, Deroceras cf. reticulatum, *Helix pomatia*, *Limacus flavus*, *Monacha carascaloides*, Tandonia cf. kusceri, *Xerolenta obvia*
3	44.09339 27.06393	village Srebarna, meadows near roads in the village	12-15.06.2014	*Cepaea vindobonensis*, *Helix pomatia*, *Monacha carascaloides*, *Xerolenta obvia*
4	44.10314 27.06285	Srebarna Reserve, buffer zone, meadows and bushes	13.06.2014	*Helix pomatia*, *Monacha carascaloides*, *Xerolenta obvia*
5	44.10302 27.06354	Srebarna Reserve, buffer zone, meadow near lake	13.06.2014	*Cepaea vindobonensis*, *Helix pomatia*, *Monacha carascaloides*
6	44.10738 27.05965	Srebarna Reserve, buffer zone, meadow and bushes	13.06.2014	*Cepaea vindobonensis*, *Helix pomatia*, *Monacha carascaloides*
7	44.10758 27.05853	Srebarna Reserve, buffer zone, meadow and bushes	13.06.2014	*Cepaea vindobonensis*
8	44.11252 27.05658	Srebarna Reserve, buffer zone, meadow and bushes	13.06.2014	*Helix pomatia*, *Monacha carascaloides*
9	44.12067 27.06315	Srebarna Reserve, buffer zone, meadow	13.06.2014	*Zebrina detrita*
10	44.13043 27.06131	Srebarna Reserve, buffer zone, open terrain near Danube	13.06.2014	*Cepaea vindobonensis*, *Helix pomatia*
11	44.12875 27.06027	Srebarna Reserve, buffer zone, forest	13.06.2014	*Acanthinula aculeata*, *Cecilioides acicula*, *Cepaea vindobonensis*, *Chondrula microtragus*, *Helix pomatia*, *Laciniaria plicata*, *Lindholmiola girva*, *Monacha carascaloides*, Oxyloma cf. elegans, *Pomatias elegans*, *Punctum pygmaeum*, *Trochulus hispidus*, *Truncatellina cylindrica*, *Truncatellina rothi*, *Vallonia costata*, *Vallonia enniensis*, *Vallonia excentrica*, *Vallonia pulchella*, *Xerolenta obvia*, *Zebrina detrita*
12	44.08833 27.06701	Srebarna Reserve, buffer zone, meadow and bushes	14.06.2014	*Cepaea vindobonensis*, *Helix pomatia*, *Monacha carascaloides*, *Xerolenta obvia*
13	44.08865 27.06411	Srebarna Reserve, buffer zone, meadow and bushes	14.06.2014	*Cepaea vindobonensis*, *Helix pomatia*, *Monacha carascaloides*, *Xerolenta obvia*
14	44.08939 27.07399	Srebarna Reserve, buffer zone, meadow and bushes	14.06.2014	*Cepaea vindobonensis*, *Helix pomatia*, *Monacha carascaloides*, *Xerolenta obvia*
15	44.08937 27.06819	Srebarna Reserve, buffer zone, forest	14.06.2014	*Chondrula tridens*, Tandonia cf. kusceri, *Truncatellina cylindrica*, *Vallonia costata*, Vallonia cf. excentrica, *Vallonia pulchella*
16	44.08802 27.06490	Srebarna Reserve, buffer zone, cane near the water, single acacias	19.07.2014	*Monachoides incarnatus*, Oxyloma cf. elegans, Tandonia cf. kusceri
17	44.09689 27.09480	Srebarna Reserve, buffer zone, shrubs near road, deciduous forest ecotone	19.07.2014	*Pomatias elegans*
18	44.09999 27.09350	Srebarna Reserve, buffer zone, open terrain near gazebo	19.07.2014	*Chondrula microtragus*
19	44.10249 27.09153	Srebarna Reserve, buffer zone, acacias' forest	19.07.2014	*Aegopinella minor*, *Lindholmiola girva*, *Oxychilus* sp. (only juvenile specimens)
20	44.10766 27.08478	Srebarna Reserve, buffer zone, coniferous forest (Pinus nigra)	19.07.2014	*Aegopinella minor*
21	44.11780 27.0797	Srebarna Reserve, buffer zone, shrubs	19.07.2014	*Zebrina detrita*
22	44.13167 27.08780	Srebarna Reserve, buffer zone, bank of the Danube river, northern Gateway	19.07.2014	Oxyloma cf. elegans
23	44.09623 27.07008	Srebarna Reserve, buffer zone, grasses near lake shore (near the village)	20.07.2014	*Chondrula tridens*
